# Bis[*O*,*O*′-bis­(4-*tert*-butyl­phen­yl) di­thio­phosphato-κ^2^ *S*,*S*′]bis­(pyridine-κ*N*)lead(II)

**DOI:** 10.1107/S1600536813023945

**Published:** 2013-08-31

**Authors:** Xiulan Zhang, Bin Xie, Linxin He, Lu Lu, Neng Chen

**Affiliations:** a Institute of Functionalized Materials, Sichuan University of Science and Engineering, Zigong 643000, People’s Republic of China; b College of Chemistry and Pharmaceutical Engineering, Sichuan University of Science and Engineering, Zigong 643000, People’s Republic of China

## Abstract

In the title compound, [Pb(C_20_H_26_O_2_PS_2_)_2_(C_5_H_5_N)_2_], the Pb^II^ ion is coordinated by two *S*,*S*′-bidentate anions and two pyridine mol­ecules. The PbN_2_S_4_ coordination geometry approximates to a penta­gonal bipyramid with one equatorial site vacant. The N atoms occupy the axial sites. One of the pyridine mol­ecules is disordered over two sets of sites in a 0.907 (7):0.093 (7) ratio and one of the *tert*-butyl groups is disordered over two sets of sites in a 0.534 (6):0.466 (6) ratio. An intra­molecular C—H⋯O inter­action occurs in one of the ligands. In the crystal, pairs of short Pb⋯S contacts [3.4018 (11) Å] generate a centrosymmetric dimeric assembly with the distant S atom lying in the region of the vacant coordination site of the metal atom. No directional packing inter­actions occur.

## Related literature

For the preparation of the ligand, see: Li & Xie (1997 ▶). For van der Waals radii, see: Bondi (1964 ▶).
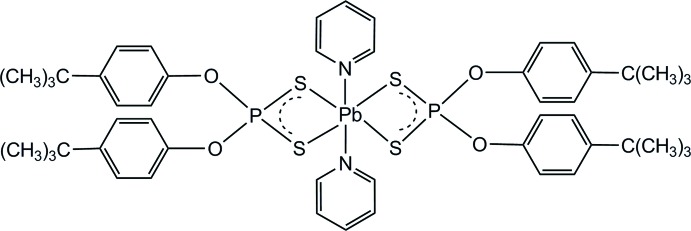


## Experimental

### 

#### Crystal data

[Pb(C_20_H_26_O_2_PS_2_)_2_(C_5_H_5_N)_2_]
*M* *_r_* = 1152.39
Triclinic, 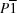

*a* = 12.4260 (3) Å
*b* = 12.9136 (3) Å
*c* = 17.9749 (4) Å
α = 89.7528 (18)°
β = 79.4467 (19)°
γ = 71.298 (2)°
*V* = 2681.22 (10) Å^3^
*Z* = 2
Mo *K*α radiation
μ = 3.40 mm^−1^
*T* = 150 K
0.25 × 0.20 × 0.20 mm

#### Data collection

Bruker APEXII diffractometer
Absorption correction: multi-scan (*SADABS*; Bruker, 2008 ▶) *T*_min_ = 0.483, *T*_max_ = 0.549
22556 measured reflections
10953 independent reflections
9252 reflections with *I* > 2σ(*I*)
*R*_int_ = 0.031

#### Refinement

*R*[*F* ^2^ > 2σ(*F* ^2^)] = 0.033
*wR*(*F* ^2^) = 0.068
*S* = 1.02
10953 reflections
663 parameters
572 restraints
H-atom parameters constrained
Δρ_max_ = 0.80 e Å^−3^
Δρ_min_ = −0.69 e Å^−3^

### 

Data collection: *APEX2* (Bruker, 2008 ▶); cell refinement: *SAINT* (Bruker, 2008 ▶); data reduction: *SAINT*; program(s) used to solve structure: *SHELXS97* (Sheldrick, 2008 ▶); program(s) used to refine structure: *SHELXL97* (Sheldrick, 2008 ▶); molecular graphics: *SHELXTL* (Sheldrick, 2008 ▶); software used to prepare material for publication: *SHELXTL*.

## Supplementary Material

Crystal structure: contains datablock(s) I. DOI: 10.1107/S1600536813023945/hb7129sup1.cif

Structure factors: contains datablock(s) I. DOI: 10.1107/S1600536813023945/hb7129Isup2.hkl

Additional supplementary materials:  crystallographic information; 3D view; checkCIF report

## Figures and Tables

**Table 1:** Selected bond lengths (Å)

Pb1—N1	2.711 (3)
Pb1—N2	2.732 (18)
Pb1—S3	2.8090 (9)
Pb1—S1	2.9009 (9)
Pb1—S4	3.0577 (9)
Pb1—S2	3.0742 (9)

**Table 2:** Hydrogen-bond geometry (Å, °)

*D*—H⋯*A*	*D*—H	H⋯*A*	*D*⋯*A*	*D*—H⋯*A*
C12—H12⋯O1	0.95	2.45	3.083 (5)	124

**Fig. 1.**
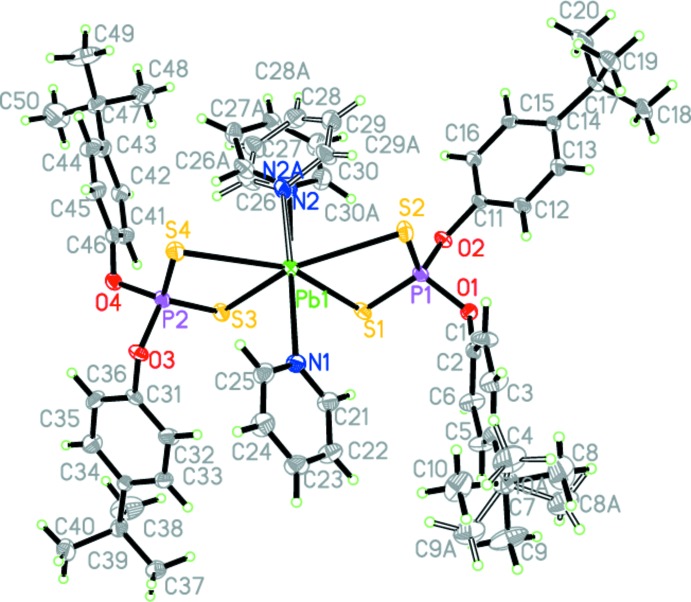
The crystal structure with displacement ellipsoids shown at the 30% level.

## supplementary crystallographic information

### 1. Comment

The crystal structure of the title compound is presented herein. The asymmetric unit contains Pb(II)cation, two C_20_H_26_O_2_PS_2_ ligand anions, two pyridine molecule. The local coordination environment around Pb(II) centers is depicted in Fig. 1.

The Pb(II) ion is coordinated by four S atoms of two bis(4-tert-butylphenyl) dithiophosphate anion ligands with Pb···S distances from 2.8089 (9) to 3.0742 (9)\%A, and two N atoms of two pyridine molecules. The PbN_2_S_4_ coordination geometry approximates to a pentagonal bipyramid with one equatorial site vacant. The N atoms occupy the axial sites.

In the crystal, short Pb···S contacts [3.4018 (11) Å] generate a dimeric assembly. The Bondi (1964) contact distance for Pb and S is 3.80Å. No directional contacts could be identified in the packing.

### 2. Experimental

bis(4-tert-Butylphenyl) dithiophosphate (L) was synthesized according to the procedure described by Li and Xie (1997). The compound PbL_2_ was prepared by treatment of Pb(NO_3_)_2_ (0.33 g, 1.0 mmol) with L (0.94 g, 2.0 mmol) in methanol (40 ml). After refluxing for 3 h, the resulting mixture was cooled to room temperature, the precipitate was filtered off, washed with methanol, and the product PbL_2_ was obtained as a colorless solid. The product was treated with pyridine (0.51 g, 6.5 mmol) in refluxing acetone and methanol (v/v = 1:1) solution for 5 h. the solution was cooled to room temperature, the precipitate was filtered off, and the adduct [PbL_2_(Py)_2_] was obtained as a colorless solid. The filtrate was slowly evaporated at room temperature for several days until colorless blocks of the title adduct appeared.

### Crystal data

**Table:**

[Pb(C_20_H_26_O_2_PS_2_)_2_(C_5_H_5_N)_2_]	*Z* = 2
*M**_r_* = 1152.39	*F*(000) = 1168
Triclinic, *P*1	*D*_x_ = 1.427 Mg m^−^^3^
Hall symbol: -P 1	Mo *K*α radiation, λ = 0.71073 Å
*a* = 12.4260 (3) Å	Cell parameters from 10707 reflections
*b* = 12.9136 (3) Å	θ = 2.9–29.2°
*c* = 17.9749 (4) Å	µ = 3.40 mm^−^^1^
α = 89.7528 (18)°	*T* = 150 K
β = 79.4467 (19)°	Block, colorless
γ = 71.298 (2)°	0.25 × 0.20 × 0.20 mm
*V* = 2681.22 (10) Å^3^	

### Data collection

**Table:**

Bruker APEXII diffractometer	10953 independent reflections
Radiation source: fine-focus sealed tube	9252 reflections with *I* > 2σ(*I*)
Graphite monochromator	*R*_int_ = 0.031
ω scans	θ_max_ = 26.4°, θ_min_ = 2.9°
Absorption correction: multi-scan (*SADABS*; Bruker, 2008)	*h* = −15→15
*T*_min_ = 0.483, *T*_max_ = 0.549	*k* = −16→13
22556 measured reflections	*l* = −22→22

### Refinement

**Table:**

Refinement on *F*^2^	Primary atom site location: structure-invariant direct methods
Least-squares matrix: full	Secondary atom site location: difference Fourier map
*R*[*F*^2^ > 2σ(*F*^2^)] = 0.033	Hydrogen site location: inferred from neighbouring sites
*wR*(*F*^2^) = 0.068	H-atom parameters constrained
*S* = 1.02	*w* = 1/[σ^2^(*F*_o_^2^) + (0.025*P*)^2^ + 0.850*P*] where *P* = (*F*_o_^2^ + 2*F*_c_^2^)/3
10953 reflections	(Δ/σ)_max_ = 0.002
663 parameters	Δρ_max_ = 0.80 e Å^−^^3^
572 restraints	Δρ_min_ = −0.69 e Å^−^^3^

### Special details

**Table:**

Geometry. All e.s.d.'s (except the e.s.d. in the dihedral angle between two l.s. planes) are estimated using the full covariance matrix. The cell e.s.d.'s are taken into account individually in the estimation of e.s.d.'s in distances, angles and torsion angles; correlations between e.s.d.'s in cell parameters are only used when they are defined by crystal symmetry. An approximate (isotropic) treatment of cell e.s.d.'s is used for estimating e.s.d.'s involving l.s. planes.
Refinement. Refinement of *F*^2^ against ALL reflections. The weighted *R*-factor *wR* and goodness of fit *S* are based on *F*^2^, conventional *R*-factors *R* are based on *F*, with *F* set to zero for negative *F*^2^. The threshold expression of *F*^2^ > σ(*F*^2^) is used only for calculating *R*-factors(gt) *etc*. and is not relevant to the choice of reflections for refinement. *R*-factors based on *F*^2^ are statistically about twice as large as those based on *F*, and *R*- factors based on ALL data will be even larger.

### Fractional atomic coordinates and isotropic or equivalent isotropic displacement parameters (Å^2^)

**Table:**

	*x*	*y*	*z*	*U*_iso_*/*U*_eq_	Occ. (<1)
Pb1	0.612885 (11)	0.604091 (11)	0.396926 (7)	0.03145 (5)	
P1	0.62254 (8)	0.66393 (7)	0.58583 (5)	0.0271 (2)	
P2	0.70541 (8)	0.61341 (7)	0.20625 (5)	0.0282 (2)	
S1	0.72725 (8)	0.69068 (7)	0.49614 (5)	0.0335 (2)	
S2	0.51198 (9)	0.59419 (8)	0.56523 (5)	0.0404 (2)	
S3	0.74784 (8)	0.68922 (8)	0.28674 (5)	0.0341 (2)	
S4	0.57837 (8)	0.55505 (8)	0.23833 (5)	0.0371 (2)	
O1	0.6962 (2)	0.60457 (18)	0.64789 (13)	0.0333 (6)	
O2	0.55836 (19)	0.78039 (17)	0.63186 (12)	0.0315 (6)	
O3	0.81414 (19)	0.51538 (18)	0.16392 (13)	0.0333 (6)	
O4	0.6861 (2)	0.69140 (18)	0.13647 (12)	0.0324 (6)	
N1	0.7945 (3)	0.4160 (2)	0.38507 (17)	0.0363 (7)	
C1	0.7648 (3)	0.4943 (3)	0.64080 (18)	0.0288 (8)	
C2	0.7151 (3)	0.4137 (3)	0.6512 (3)	0.0522 (12)	
H2	0.6333	0.4316	0.6588	0.063*	
C3	0.7845 (4)	0.3060 (3)	0.6505 (3)	0.0567 (12)	
H3	0.7490	0.2506	0.6567	0.068*	
C4	0.9035 (3)	0.2758 (3)	0.6412 (2)	0.0354 (9)	
C5	0.9505 (3)	0.3594 (3)	0.6319 (2)	0.0469 (11)	
H5	1.0320	0.3423	0.6261	0.056*	
C6	0.8818 (3)	0.4681 (3)	0.6309 (2)	0.0436 (10)	
H6	0.9166	0.5239	0.6234	0.052*	
C7	0.9755 (3)	0.1559 (3)	0.6426 (2)	0.0474 (10)	
C8	0.9290 (7)	0.1076 (4)	0.7139 (3)	0.099 (3)	0.907 (7)
H8A	0.9772	0.0312	0.7153	0.148*	0.907 (7)
H8B	0.8492	0.1109	0.7135	0.148*	0.907 (7)
H8C	0.9306	0.1496	0.7587	0.148*	0.907 (7)
C9	1.1030 (5)	0.1402 (4)	0.6397 (5)	0.089 (2)	0.907 (7)
H9A	1.1454	0.0623	0.6420	0.133*	0.907 (7)
H9B	1.1110	0.1813	0.6829	0.133*	0.907 (7)
H9C	1.1346	0.1671	0.5924	0.133*	0.907 (7)
C10	0.9666 (6)	0.0917 (4)	0.5746 (3)	0.0732 (19)	0.907 (7)
H10A	1.0131	0.0146	0.5756	0.110*	0.907 (7)
H10B	0.9956	0.1217	0.5278	0.110*	0.907 (7)
H10C	0.8856	0.0976	0.5764	0.110*	0.907 (7)
C8A	1.014 (6)	0.151 (4)	0.719 (2)	0.089 (10)	0.093 (7)
H8D	1.0485	0.0746	0.7299	0.133*	0.093 (7)
H8E	0.9474	0.1874	0.7586	0.133*	0.093 (7)
H8F	1.0720	0.1887	0.7162	0.133*	0.093 (7)
C9A	1.089 (3)	0.135 (5)	0.587 (3)	0.083 (11)	0.093 (7)
H9D	1.0738	0.1472	0.5354	0.125*	0.093 (7)
H9E	1.1385	0.0588	0.5890	0.125*	0.093 (7)
H9F	1.1289	0.1846	0.6001	0.125*	0.093 (7)
C10A	0.898 (4)	0.086 (3)	0.644 (4)	0.095 (11)	0.093 (7)
H10D	0.8238	0.1295	0.6311	0.142*	0.093 (7)
H10E	0.8833	0.0592	0.6950	0.142*	0.093 (7)
H10F	0.9354	0.0231	0.6073	0.142*	0.093 (7)
C11	0.4633 (3)	0.7995 (3)	0.69197 (18)	0.0268 (7)	
C12	0.4578 (3)	0.7252 (3)	0.7470 (2)	0.0372 (9)	
H12	0.5172	0.6563	0.7437	0.045*	
C13	0.3648 (3)	0.7526 (3)	0.8070 (2)	0.0370 (9)	
H13	0.3610	0.7013	0.8446	0.044*	
C14	0.2771 (3)	0.8520 (3)	0.81404 (19)	0.0316 (8)	
C15	0.2841 (3)	0.9232 (3)	0.75728 (19)	0.0321 (8)	
H15	0.2241	0.9916	0.7601	0.038*	
C16	0.3763 (3)	0.8975 (3)	0.69617 (19)	0.0304 (8)	
H16	0.3789	0.9477	0.6576	0.037*	
C17	0.1796 (3)	0.8792 (3)	0.8847 (2)	0.0424 (10)	
C18	0.2339 (4)	0.8845 (4)	0.9543 (2)	0.0606 (13)	
H18C	0.2695	0.9424	0.9492	0.091*	
H18A	0.2932	0.8140	0.9577	0.091*	
H18B	0.1738	0.9004	1.0002	0.091*	
C19	0.1218 (4)	0.7908 (4)	0.8933 (3)	0.0632 (13)	
H19C	0.0615	0.8082	0.9392	0.095*	
H19B	0.1798	0.7198	0.8972	0.095*	
H19A	0.0869	0.7877	0.8490	0.095*	
C20	0.0872 (4)	0.9891 (4)	0.8809 (3)	0.0696 (15)	
H20C	0.1223	1.0473	0.8776	0.104*	
H20B	0.0267	1.0032	0.9266	0.104*	
H20A	0.0530	0.9875	0.8361	0.104*	
C21	0.8799 (3)	0.4028 (3)	0.4228 (2)	0.0448 (10)	
H21	0.8759	0.4607	0.4568	0.054*	
C22	0.9744 (4)	0.3083 (3)	0.4147 (2)	0.0492 (11)	
H22	1.0344	0.3014	0.4423	0.059*	
C23	0.9798 (4)	0.2249 (3)	0.3660 (2)	0.0502 (11)	
H23	1.0439	0.1591	0.3588	0.060*	
C24	0.8910 (4)	0.2380 (3)	0.3278 (2)	0.0530 (11)	
H24	0.8925	0.1810	0.2940	0.064*	
C25	0.7997 (4)	0.3342 (3)	0.3387 (2)	0.0444 (10)	
H25	0.7383	0.3424	0.3122	0.053*	
N2	0.4568 (16)	0.8110 (13)	0.4130 (4)	0.051 (3)	0.534 (6)
C26	0.4243 (9)	0.8513 (7)	0.3507 (5)	0.047 (3)	0.534 (6)
H26	0.4517	0.8029	0.3066	0.056*	0.534 (6)
C27	0.3548 (7)	0.9559 (6)	0.3418 (5)	0.0426 (19)	0.534 (6)
H27	0.3262	0.9765	0.2965	0.051*	0.534 (6)
C28	0.3301 (8)	1.0278 (7)	0.4026 (4)	0.045 (2)	0.534 (6)
H28	0.2818	1.1013	0.4012	0.054*	0.534 (6)
C29	0.3750 (8)	0.9935 (6)	0.4654 (5)	0.061 (2)	0.534 (6)
H29	0.3588	1.0420	0.5087	0.073*	0.534 (6)
C30	0.4437 (9)	0.8876 (6)	0.4643 (5)	0.067 (3)	0.534 (6)
H30	0.4857	0.8675	0.5041	0.081*	0.534 (6)
N2A	0.460 (2)	0.7957 (17)	0.4024 (6)	0.054 (3)	0.466 (6)
C26A	0.4523 (12)	0.8674 (9)	0.3542 (6)	0.061 (3)	0.466 (6)
H26A	0.4936	0.8452	0.3039	0.073*	0.466 (6)
C27A	0.3878 (9)	0.9755 (8)	0.3700 (6)	0.057 (3)	0.466 (6)
H27A	0.3932	1.0264	0.3324	0.069*	0.466 (6)
C28A	0.3161 (10)	1.0120 (8)	0.4382 (6)	0.057 (3)	0.466 (6)
H28A	0.2724	1.0873	0.4490	0.068*	0.466 (6)
C29A	0.3097 (10)	0.9362 (7)	0.4902 (5)	0.070 (3)	0.466 (6)
H29A	0.2588	0.9577	0.5379	0.084*	0.466 (6)
C30A	0.3765 (9)	0.8293 (7)	0.4737 (5)	0.064 (3)	0.466 (6)
H30A	0.3687	0.7760	0.5090	0.077*	0.466 (6)
C31	0.9261 (3)	0.5243 (3)	0.14579 (19)	0.0291 (8)	
C32	1.0056 (3)	0.4731 (3)	0.1880 (2)	0.0440 (10)	
H32	0.9846	0.4345	0.2304	0.053*	
C33	1.1175 (3)	0.4770 (3)	0.1691 (2)	0.0425 (10)	
H33	1.1726	0.4411	0.1991	0.051*	
C34	1.1505 (3)	0.5319 (3)	0.10792 (19)	0.0307 (8)	
C35	1.0673 (3)	0.5826 (4)	0.0672 (2)	0.0530 (12)	
H35	1.0877	0.6214	0.0248	0.064*	
C36	0.9545 (3)	0.5803 (4)	0.0848 (2)	0.0509 (11)	
H36	0.8989	0.6165	0.0553	0.061*	
C37	1.2725 (3)	0.5398 (3)	0.0861 (2)	0.0392 (9)	
C38	1.3553 (3)	0.4724 (4)	0.1342 (2)	0.0578 (12)	
H38B	1.3263	0.4985	0.1875	0.087*	
H38C	1.4319	0.4800	0.1174	0.087*	
H38A	1.3612	0.3952	0.1286	0.087*	
C39	1.3226 (4)	0.4988 (5)	0.0037 (2)	0.0761 (16)	
H39C	1.4022	0.5000	−0.0095	0.114*	
H39B	1.2755	0.5462	−0.0292	0.114*	
H39A	1.3222	0.4237	−0.0033	0.114*	
C40	1.2644 (4)	0.6592 (4)	0.0955 (4)	0.0893 (19)	
H40C	1.3401	0.6670	0.0757	0.134*	
H40B	1.2407	0.6833	0.1494	0.134*	
H40A	1.2071	0.7042	0.0676	0.134*	
C41	0.5936 (3)	0.7898 (3)	0.14164 (18)	0.0314 (8)	
C42	0.4882 (3)	0.7893 (3)	0.1280 (2)	0.0445 (10)	
H42	0.4760	0.7219	0.1191	0.053*	
C43	0.4006 (3)	0.8869 (3)	0.1275 (2)	0.0473 (10)	
H43	0.3283	0.8853	0.1181	0.057*	
C44	0.4144 (3)	0.9882 (3)	0.1401 (2)	0.0377 (9)	
C45	0.5218 (3)	0.9850 (3)	0.1545 (2)	0.0376 (9)	
H45	0.5346	1.0520	0.1640	0.045*	
C46	0.6104 (3)	0.8876 (3)	0.1554 (2)	0.0367 (9)	
H46	0.6826	0.8883	0.1654	0.044*	
C47	0.3209 (3)	1.0973 (3)	0.1363 (2)	0.0459 (10)	
C48	0.2985 (4)	1.1711 (4)	0.2073 (3)	0.0787 (16)	
H48C	0.3704	1.1839	0.2128	0.118*	
H48A	0.2715	1.1357	0.2519	0.118*	
H48B	0.2394	1.2413	0.2029	0.118*	
C49	0.2050 (4)	1.0846 (4)	0.1314 (4)	0.090 (2)	
H49C	0.1457	1.1569	0.1363	0.135*	
H49A	0.1838	1.0403	0.1724	0.135*	
H49B	0.2108	1.0482	0.0824	0.135*	
C50	0.3605 (5)	1.1537 (4)	0.0675 (3)	0.093 (2)	
H50C	0.4339	1.1644	0.0714	0.139*	
H50B	0.3018	1.2249	0.0650	0.139*	
H50A	0.3714	1.1081	0.0215	0.139*	

### Atomic displacement parameters (Å^2^)

**Table:**

	*U*^11^	*U*^22^	*U*^33^	*U*^12^	*U*^13^	*U*^23^
Pb1	0.02567 (8)	0.03976 (9)	0.02520 (7)	−0.00793 (6)	−0.00047 (5)	−0.00004 (5)
P1	0.0258 (5)	0.0289 (5)	0.0254 (4)	−0.0084 (4)	−0.0026 (4)	−0.0029 (4)
P2	0.0242 (5)	0.0363 (5)	0.0259 (4)	−0.0153 (4)	0.0009 (4)	−0.0022 (4)
S1	0.0294 (5)	0.0375 (5)	0.0311 (5)	−0.0134 (4)	0.0050 (4)	−0.0050 (4)
S2	0.0445 (6)	0.0620 (6)	0.0262 (5)	−0.0345 (5)	−0.0036 (4)	−0.0003 (4)
S3	0.0318 (5)	0.0459 (5)	0.0292 (5)	−0.0206 (5)	−0.0029 (4)	−0.0043 (4)
S4	0.0322 (5)	0.0540 (6)	0.0345 (5)	−0.0282 (5)	−0.0039 (4)	0.0012 (4)
O1	0.0335 (14)	0.0305 (13)	0.0288 (13)	−0.0003 (12)	−0.0067 (11)	−0.0083 (10)
O2	0.0290 (13)	0.0284 (13)	0.0311 (13)	−0.0064 (11)	0.0042 (11)	−0.0027 (10)
O3	0.0240 (13)	0.0360 (14)	0.0377 (14)	−0.0115 (11)	0.0035 (11)	−0.0056 (11)
O4	0.0307 (14)	0.0380 (14)	0.0283 (13)	−0.0133 (12)	−0.0011 (11)	0.0017 (10)
N1	0.0330 (17)	0.0361 (17)	0.0383 (17)	−0.0093 (15)	−0.0062 (15)	−0.0019 (14)
C1	0.0293 (19)	0.0299 (19)	0.0233 (17)	−0.0065 (17)	−0.0008 (15)	0.0004 (14)
C2	0.025 (2)	0.047 (3)	0.080 (3)	−0.012 (2)	0.000 (2)	0.020 (2)
C3	0.047 (3)	0.045 (3)	0.089 (4)	−0.027 (2)	−0.020 (3)	0.027 (2)
C4	0.038 (2)	0.0295 (19)	0.041 (2)	−0.0122 (18)	−0.0110 (18)	0.0043 (16)
C5	0.028 (2)	0.035 (2)	0.078 (3)	−0.0089 (19)	−0.015 (2)	0.000 (2)
C6	0.036 (2)	0.028 (2)	0.074 (3)	−0.0164 (19)	−0.018 (2)	0.0041 (19)
C7	0.053 (3)	0.031 (2)	0.061 (3)	−0.013 (2)	−0.019 (2)	0.0058 (19)
C8	0.141 (6)	0.045 (3)	0.081 (4)	−0.001 (4)	−0.003 (4)	0.032 (3)
C9	0.071 (4)	0.035 (3)	0.163 (7)	−0.002 (3)	−0.058 (4)	0.014 (4)
C10	0.106 (5)	0.033 (3)	0.080 (4)	−0.008 (3)	−0.042 (4)	−0.006 (3)
C8A	0.095 (17)	0.039 (15)	0.106 (16)	0.015 (15)	−0.023 (16)	0.022 (15)
C9A	0.089 (16)	0.036 (15)	0.102 (17)	0.014 (15)	−0.023 (16)	0.012 (16)
C10A	0.106 (16)	0.039 (15)	0.110 (17)	0.019 (15)	−0.027 (16)	0.021 (15)
C11	0.0235 (18)	0.0301 (19)	0.0246 (17)	−0.0075 (16)	−0.0010 (15)	−0.0035 (14)
C12	0.031 (2)	0.032 (2)	0.038 (2)	0.0011 (17)	0.0005 (17)	0.0014 (16)
C13	0.037 (2)	0.041 (2)	0.0293 (19)	−0.0083 (19)	−0.0040 (17)	0.0083 (16)
C14	0.0256 (19)	0.043 (2)	0.0248 (18)	−0.0087 (18)	−0.0054 (15)	−0.0029 (15)
C15	0.0249 (19)	0.0334 (19)	0.0313 (19)	0.0001 (16)	−0.0065 (16)	−0.0016 (15)
C16	0.0293 (19)	0.0303 (19)	0.0291 (18)	−0.0068 (17)	−0.0042 (16)	−0.0004 (15)
C17	0.029 (2)	0.058 (3)	0.031 (2)	−0.005 (2)	0.0021 (17)	0.0012 (18)
C18	0.052 (3)	0.097 (4)	0.026 (2)	−0.021 (3)	0.004 (2)	−0.011 (2)
C19	0.046 (3)	0.092 (4)	0.051 (3)	−0.031 (3)	0.006 (2)	0.009 (2)
C20	0.043 (3)	0.078 (3)	0.055 (3)	0.010 (3)	0.019 (2)	0.001 (2)
C21	0.042 (2)	0.041 (2)	0.050 (2)	−0.009 (2)	−0.016 (2)	−0.0088 (19)
C22	0.045 (3)	0.041 (2)	0.065 (3)	−0.011 (2)	−0.024 (2)	0.002 (2)
C23	0.050 (3)	0.030 (2)	0.062 (3)	0.000 (2)	−0.013 (2)	−0.0028 (19)
C24	0.062 (3)	0.042 (2)	0.058 (3)	−0.015 (2)	−0.021 (2)	−0.011 (2)
C25	0.044 (2)	0.046 (2)	0.046 (2)	−0.013 (2)	−0.016 (2)	−0.0020 (19)
N2	0.053 (4)	0.045 (5)	0.027 (4)	0.023 (4)	−0.009 (4)	−0.020 (3)
C26	0.049 (5)	0.034 (4)	0.045 (4)	−0.010 (4)	0.011 (4)	0.000 (3)
C27	0.056 (4)	0.035 (4)	0.037 (4)	−0.014 (3)	−0.010 (3)	−0.005 (3)
C28	0.046 (4)	0.040 (4)	0.040 (4)	−0.008 (3)	0.002 (4)	−0.001 (4)
C29	0.073 (5)	0.042 (4)	0.045 (4)	0.017 (4)	−0.020 (4)	−0.012 (3)
C30	0.074 (5)	0.050 (4)	0.046 (4)	0.025 (4)	−0.015 (4)	−0.010 (3)
N2A	0.051 (5)	0.058 (6)	0.029 (4)	0.016 (5)	−0.002 (4)	−0.016 (4)
C26A	0.059 (6)	0.042 (5)	0.057 (5)	−0.001 (5)	0.021 (4)	0.010 (4)
C27A	0.058 (5)	0.042 (5)	0.059 (5)	−0.006 (4)	0.003 (4)	0.005 (4)
C28A	0.054 (5)	0.042 (4)	0.060 (5)	0.007 (4)	−0.015 (5)	−0.022 (4)
C29A	0.063 (5)	0.071 (5)	0.045 (4)	0.020 (4)	−0.006 (4)	−0.013 (4)
C30A	0.055 (5)	0.057 (5)	0.046 (4)	0.017 (4)	0.012 (4)	0.011 (4)
C31	0.0230 (18)	0.0324 (19)	0.0295 (18)	−0.0109 (16)	0.0047 (15)	−0.0047 (15)
C32	0.033 (2)	0.045 (2)	0.046 (2)	−0.0060 (19)	0.0025 (19)	0.0185 (18)
C33	0.029 (2)	0.052 (2)	0.040 (2)	−0.0059 (19)	−0.0043 (18)	0.0130 (18)
C34	0.0274 (19)	0.036 (2)	0.0288 (18)	−0.0108 (17)	−0.0059 (16)	−0.0031 (15)
C35	0.035 (2)	0.082 (3)	0.045 (2)	−0.025 (2)	−0.004 (2)	0.028 (2)
C36	0.032 (2)	0.087 (3)	0.040 (2)	−0.023 (2)	−0.0131 (19)	0.025 (2)
C37	0.034 (2)	0.053 (2)	0.037 (2)	−0.021 (2)	−0.0105 (18)	0.0033 (18)
C38	0.028 (2)	0.095 (4)	0.050 (3)	−0.019 (2)	−0.009 (2)	0.003 (2)
C39	0.036 (3)	0.154 (5)	0.039 (3)	−0.037 (3)	0.002 (2)	0.004 (3)
C40	0.062 (3)	0.074 (4)	0.152 (6)	−0.042 (3)	−0.036 (4)	0.015 (4)
C41	0.034 (2)	0.039 (2)	0.0222 (17)	−0.0138 (18)	−0.0037 (16)	0.0013 (15)
C42	0.044 (2)	0.038 (2)	0.057 (3)	−0.016 (2)	−0.019 (2)	−0.0053 (19)
C43	0.038 (2)	0.053 (3)	0.060 (3)	−0.021 (2)	−0.018 (2)	−0.003 (2)
C44	0.039 (2)	0.046 (2)	0.033 (2)	−0.019 (2)	−0.0084 (18)	−0.0008 (17)
C45	0.040 (2)	0.036 (2)	0.042 (2)	−0.019 (2)	−0.0095 (18)	0.0020 (17)
C46	0.033 (2)	0.046 (2)	0.039 (2)	−0.0221 (19)	−0.0090 (17)	0.0037 (17)
C47	0.039 (2)	0.049 (2)	0.052 (3)	−0.013 (2)	−0.018 (2)	0.001 (2)
C48	0.071 (4)	0.062 (3)	0.084 (4)	0.011 (3)	−0.024 (3)	−0.020 (3)
C49	0.048 (3)	0.063 (3)	0.163 (6)	−0.009 (3)	−0.046 (4)	−0.009 (4)
C50	0.096 (4)	0.070 (4)	0.083 (4)	0.002 (3)	−0.002 (4)	0.033 (3)

### Geometric parameters (Å, º)

**Table:**

Pb1—N2A	2.58 (2)	C20—H20A	0.9800
Pb1—N1	2.711 (3)	C21—C22	1.382 (5)
Pb1—N2	2.732 (18)	C21—H21	0.9500
Pb1—S3	2.8090 (9)	C22—C23	1.369 (5)
Pb1—S1	2.9009 (9)	C22—H22	0.9500
Pb1—S4	3.0577 (9)	C23—C24	1.369 (6)
Pb1—S2	3.0742 (9)	C23—H23	0.9500
P1—O2	1.607 (2)	C24—C25	1.373 (5)
P1—O1	1.608 (3)	C24—H24	0.9500
P1—S2	1.9540 (13)	C25—H25	0.9500
P1—S1	1.9816 (12)	N2—C30	1.305 (11)
P2—O3	1.594 (2)	N2—C26	1.309 (12)
P2—O4	1.609 (2)	C26—C27	1.377 (6)
P2—S4	1.9541 (12)	C26—H26	0.9500
P2—S3	1.9880 (12)	C27—C28	1.362 (6)
O1—C1	1.397 (4)	C27—H27	0.9500
O2—C11	1.404 (4)	C28—C29	1.360 (7)
O3—C31	1.411 (4)	C28—H28	0.9500
O4—C41	1.403 (4)	C29—C30	1.359 (6)
N1—C25	1.326 (4)	C29—H29	0.9500
N1—C21	1.328 (5)	C30—H30	0.9500
C1—C6	1.361 (5)	N2A—C26A	1.26 (2)
C1—C2	1.368 (5)	N2A—C30A	1.463 (19)
C2—C3	1.380 (5)	C26A—C27A	1.369 (7)
C2—H2	0.9500	C26A—H26A	0.9500
C3—C4	1.382 (5)	C27A—C28A	1.361 (7)
C3—H3	0.9500	C27A—H27A	0.9500
C4—C5	1.379 (5)	C28A—C29A	1.362 (7)
C4—C7	1.522 (5)	C28A—H28A	0.9500
C5—C6	1.391 (5)	C29A—C30A	1.364 (7)
C5—H5	0.9500	C29A—H29A	0.9500
C6—H6	0.9500	C30A—H30A	0.9500
C7—C10	1.520 (5)	C31—C32	1.356 (5)
C7—C10A	1.521 (9)	C31—C36	1.366 (5)
C7—C8	1.522 (5)	C32—C33	1.387 (5)
C7—C9A	1.522 (9)	C32—H32	0.9500
C7—C9	1.522 (5)	C33—C34	1.377 (5)
C7—C8A	1.524 (9)	C33—H33	0.9500
C8—H8A	0.9800	C34—C35	1.368 (5)
C8—H8B	0.9800	C34—C37	1.531 (5)
C8—H8C	0.9800	C35—C36	1.389 (5)
C9—H9A	0.9800	C35—H35	0.9500
C9—H9B	0.9800	C36—H36	0.9500
C9—H9C	0.9800	C37—C38	1.518 (5)
C10—H10A	0.9800	C37—C40	1.520 (6)
C10—H10B	0.9800	C37—C39	1.524 (5)
C10—H10C	0.9800	C38—H38B	0.9800
C8A—H8D	0.9800	C38—H38C	0.9800
C8A—H8E	0.9800	C38—H38A	0.9800
C8A—H8F	0.9800	C39—H39C	0.9800
C9A—H9D	0.9800	C39—H39B	0.9800
C9A—H9E	0.9800	C39—H39A	0.9800
C9A—H9F	0.9800	C40—H40C	0.9800
C10A—H10D	0.9800	C40—H40B	0.9800
C10A—H10E	0.9800	C40—H40A	0.9800
C10A—H10F	0.9800	C41—C46	1.376 (5)
C11—C16	1.368 (4)	C41—C42	1.377 (5)
C11—C12	1.384 (5)	C42—C43	1.377 (5)
C12—C13	1.381 (5)	C42—H42	0.9500
C12—H12	0.9500	C43—C44	1.398 (5)
C13—C14	1.381 (5)	C43—H43	0.9500
C13—H13	0.9500	C44—C45	1.393 (5)
C14—C15	1.380 (5)	C44—C47	1.522 (5)
C14—C17	1.540 (5)	C45—C46	1.383 (5)
C15—C16	1.389 (4)	C45—H45	0.9500
C15—H15	0.9500	C46—H46	0.9500
C16—H16	0.9500	C47—C50	1.517 (6)
C17—C20	1.523 (5)	C47—C49	1.519 (6)
C17—C19	1.525 (6)	C47—C48	1.525 (6)
C17—C18	1.537 (5)	C48—H48C	0.9800
C18—H18C	0.9800	C48—H48A	0.9800
C18—H18A	0.9800	C48—H48B	0.9800
C18—H18B	0.9800	C49—H49C	0.9800
C19—H19C	0.9800	C49—H49A	0.9800
C19—H19B	0.9800	C49—H49B	0.9800
C19—H19A	0.9800	C50—H50C	0.9800
C20—H20C	0.9800	C50—H50B	0.9800
C20—H20B	0.9800	C50—H50A	0.9800
			
N2A—Pb1—N1	172.4 (4)	H19C—C19—H19A	109.5
N2A—Pb1—N2	4.7 (5)	H19B—C19—H19A	109.5
N1—Pb1—N2	170.1 (3)	C17—C20—H20C	109.5
N2A—Pb1—S3	84.4 (3)	C17—C20—H20B	109.5
N1—Pb1—S3	88.79 (6)	H20C—C20—H20B	109.5
N2—Pb1—S3	85.2 (3)	C17—C20—H20A	109.5
N2A—Pb1—S1	89.1 (4)	H20C—C20—H20A	109.5
N1—Pb1—S1	86.54 (7)	H20B—C20—H20A	109.5
N2—Pb1—S1	84.7 (3)	N1—C21—C22	122.7 (4)
S3—Pb1—S1	81.43 (2)	N1—C21—H21	118.7
N2A—Pb1—S4	90.2 (3)	C22—C21—H21	118.7
N1—Pb1—S4	90.53 (7)	C23—C22—C21	118.5 (4)
N2—Pb1—S4	94.81 (19)	C23—C22—H22	120.7
S3—Pb1—S4	69.48 (2)	C21—C22—H22	120.7
S1—Pb1—S4	150.83 (2)	C22—C23—C24	118.8 (4)
N2A—Pb1—S2	87.1 (3)	C22—C23—H23	120.6
N1—Pb1—S2	96.97 (7)	C24—C23—H23	120.6
N2—Pb1—S2	84.10 (16)	C23—C24—C25	119.4 (4)
S3—Pb1—S2	148.29 (3)	C23—C24—H24	120.3
S1—Pb1—S2	67.91 (2)	C25—C24—H24	120.3
S4—Pb1—S2	141.17 (2)	N1—C25—C24	122.2 (4)
O2—P1—O1	97.61 (12)	N1—C25—H25	118.9
O2—P1—S2	111.63 (10)	C24—C25—H25	118.9
O1—P1—S2	113.50 (10)	C30—N2—C26	112.2 (14)
O2—P1—S1	106.22 (9)	C30—N2—Pb1	127.0 (10)
O1—P1—S1	109.96 (10)	C26—N2—Pb1	115.6 (8)
S2—P1—S1	116.15 (6)	N2—C26—C27	127.3 (11)
O3—P2—O4	98.63 (12)	N2—C26—H26	116.3
O3—P2—S4	107.59 (10)	C27—C26—H26	116.3
O4—P2—S4	112.64 (10)	C28—C27—C26	115.1 (9)
O3—P2—S3	111.16 (10)	C28—C27—H27	122.4
O4—P2—S3	109.05 (10)	C26—C27—H27	122.4
S4—P2—S3	116.34 (5)	C29—C28—C27	119.5 (8)
P1—S1—Pb1	90.15 (4)	C29—C28—H28	120.2
P1—S2—Pb1	85.72 (4)	C27—C28—H28	120.2
P2—S3—Pb1	89.40 (4)	C30—C29—C28	117.7 (8)
P2—S4—Pb1	83.07 (4)	C30—C29—H29	121.1
C1—O1—P1	123.7 (2)	C28—C29—H29	121.1
C11—O2—P1	123.5 (2)	N2—C30—C29	125.5 (10)
C31—O3—P2	122.0 (2)	N2—C30—H30	117.2
C41—O4—P2	122.82 (19)	C29—C30—H30	117.2
C25—N1—C21	118.3 (3)	C26A—N2A—C30A	115.2 (18)
C25—N1—Pb1	118.9 (3)	C26A—N2A—Pb1	129.0 (13)
C21—N1—Pb1	122.7 (2)	C30A—N2A—Pb1	115.6 (10)
C6—C1—C2	119.9 (3)	N2A—C26A—C27A	124.0 (13)
C6—C1—O1	119.2 (3)	N2A—C26A—H26A	118.0
C2—C1—O1	120.6 (3)	C27A—C26A—H26A	118.0
C1—C2—C3	119.5 (4)	C28A—C27A—C26A	121.8 (10)
C1—C2—H2	120.2	C28A—C27A—H27A	119.1
C3—C2—H2	120.2	C26A—C27A—H27A	119.1
C2—C3—C4	122.5 (4)	C27A—C28A—C29A	117.2 (9)
C2—C3—H3	118.7	C27A—C28A—H28A	121.4
C4—C3—H3	118.7	C29A—C28A—H28A	121.4
C5—C4—C3	116.3 (3)	C28A—C29A—C30A	119.9 (9)
C5—C4—C7	123.5 (3)	C28A—C29A—H29A	120.1
C3—C4—C7	120.2 (3)	C30A—C29A—H29A	120.1
C4—C5—C6	121.8 (4)	C29A—C30A—N2A	120.9 (12)
C4—C5—H5	119.1	C29A—C30A—H30A	119.6
C6—C5—H5	119.1	N2A—C30A—H30A	119.6
C1—C6—C5	119.9 (3)	C32—C31—C36	120.9 (3)
C1—C6—H6	120.0	C32—C31—O3	119.0 (3)
C5—C6—H6	120.0	C36—C31—O3	120.1 (3)
C10—C7—C10A	55 (3)	C31—C32—C33	119.9 (3)
C10—C7—C8	107.9 (4)	C31—C32—H32	120.1
C10A—C7—C8	56 (3)	C33—C32—H32	120.1
C10—C7—C9A	74 (3)	C34—C33—C32	121.4 (4)
C10A—C7—C9A	124 (4)	C34—C33—H33	119.3
C8—C7—C9A	138 (2)	C32—C33—H33	119.3
C10—C7—C4	109.6 (3)	C35—C34—C33	116.7 (3)
C10A—C7—C4	109.1 (19)	C35—C34—C37	120.4 (3)
C8—C7—C4	109.9 (3)	C33—C34—C37	122.9 (3)
C9A—C7—C4	108 (2)	C34—C35—C36	123.3 (4)
C10—C7—C9	107.8 (4)	C34—C35—H35	118.4
C10A—C7—C9	138.4 (19)	C36—C35—H35	118.4
C8—C7—C9	109.1 (5)	C31—C36—C35	117.9 (4)
C9A—C7—C9	38 (3)	C31—C36—H36	121.1
C4—C7—C9	112.4 (3)	C35—C36—H36	121.1
C10—C7—C8A	147 (2)	C38—C37—C40	108.8 (4)
C10A—C7—C8A	109 (4)	C38—C37—C39	107.7 (4)
C8—C7—C8A	53 (3)	C40—C37—C39	109.4 (4)
C9A—C7—C8A	102 (4)	C38—C37—C34	113.1 (3)
C4—C7—C8A	102.9 (19)	C40—C37—C34	108.5 (3)
C9—C7—C8A	64 (3)	C39—C37—C34	109.4 (3)
C7—C8—H8A	109.5	C37—C38—H38B	109.5
C7—C8—H8B	109.5	C37—C38—H38C	109.5
C7—C8—H8C	109.5	H38B—C38—H38C	109.5
C7—C9—H9A	109.5	C37—C38—H38A	109.5
C7—C9—H9B	109.5	H38B—C38—H38A	109.5
C7—C9—H9C	109.5	H38C—C38—H38A	109.5
C7—C10—H10A	109.5	C37—C39—H39C	109.5
C7—C10—H10B	109.5	C37—C39—H39B	109.5
C7—C10—H10C	109.5	H39C—C39—H39B	109.5
C7—C8A—H8D	109.5	C37—C39—H39A	109.5
C7—C8A—H8E	109.5	H39C—C39—H39A	109.5
H8D—C8A—H8E	109.5	H39B—C39—H39A	109.5
C7—C8A—H8F	109.5	C37—C40—H40C	109.5
H8D—C8A—H8F	109.5	C37—C40—H40B	109.5
H8E—C8A—H8F	109.5	H40C—C40—H40B	109.5
C7—C9A—H9D	109.5	C37—C40—H40A	109.5
C7—C9A—H9E	109.5	H40C—C40—H40A	109.5
H9D—C9A—H9E	109.5	H40B—C40—H40A	109.5
C7—C9A—H9F	109.5	C46—C41—C42	119.9 (4)
H9D—C9A—H9F	109.5	C46—C41—O4	119.8 (3)
H9E—C9A—H9F	109.5	C42—C41—O4	120.2 (3)
C7—C10A—H10D	109.5	C41—C42—C43	119.8 (4)
C7—C10A—H10E	109.5	C41—C42—H42	120.1
H10D—C10A—H10E	109.5	C43—C42—H42	120.1
C7—C10A—H10F	109.5	C42—C43—C44	122.3 (4)
H10D—C10A—H10F	109.5	C42—C43—H43	118.8
H10E—C10A—H10F	109.5	C44—C43—H43	118.8
C16—C11—C12	120.2 (3)	C45—C44—C43	116.1 (4)
C16—C11—O2	117.3 (3)	C45—C44—C47	120.5 (3)
C12—C11—O2	122.4 (3)	C43—C44—C47	123.4 (3)
C13—C12—C11	119.0 (3)	C46—C45—C44	122.3 (3)
C13—C12—H12	120.5	C46—C45—H45	118.9
C11—C12—H12	120.5	C44—C45—H45	118.9
C14—C13—C12	122.2 (3)	C41—C46—C45	119.7 (3)
C14—C13—H13	118.9	C41—C46—H46	120.1
C12—C13—H13	118.9	C45—C46—H46	120.1
C15—C14—C13	117.2 (3)	C50—C47—C49	109.3 (4)
C15—C14—C17	123.6 (3)	C50—C47—C44	108.7 (3)
C13—C14—C17	119.2 (3)	C49—C47—C44	112.7 (4)
C14—C15—C16	121.8 (3)	C50—C47—C48	109.2 (4)
C14—C15—H15	119.1	C49—C47—C48	105.7 (4)
C16—C15—H15	119.1	C44—C47—C48	111.2 (3)
C11—C16—C15	119.5 (3)	C47—C48—H48C	109.5
C11—C16—H16	120.3	C47—C48—H48A	109.5
C15—C16—H16	120.3	H48C—C48—H48A	109.5
C20—C17—C19	108.4 (4)	C47—C48—H48B	109.5
C20—C17—C18	108.6 (4)	H48C—C48—H48B	109.5
C19—C17—C18	109.5 (3)	H48A—C48—H48B	109.5
C20—C17—C14	112.0 (3)	C47—C49—H49C	109.5
C19—C17—C14	110.5 (3)	C47—C49—H49A	109.5
C18—C17—C14	107.8 (3)	H49C—C49—H49A	109.5
C17—C18—H18C	109.5	C47—C49—H49B	109.5
C17—C18—H18A	109.5	H49C—C49—H49B	109.5
H18C—C18—H18A	109.5	H49A—C49—H49B	109.5
C17—C18—H18B	109.5	C47—C50—H50C	109.5
H18C—C18—H18B	109.5	C47—C50—H50B	109.5
H18A—C18—H18B	109.5	H50C—C50—H50B	109.5
C17—C19—H19C	109.5	C47—C50—H50A	109.5
C17—C19—H19B	109.5	H50C—C50—H50A	109.5
H19C—C19—H19B	109.5	H50B—C50—H50A	109.5
C17—C19—H19A	109.5		
			
O2—P1—S1—Pb1	−122.28 (10)	C12—C11—C16—C15	1.9 (5)
O1—P1—S1—Pb1	133.11 (9)	O2—C11—C16—C15	−175.9 (3)
S2—P1—S1—Pb1	2.50 (6)	C14—C15—C16—C11	−0.4 (5)
N2A—Pb1—S1—P1	85.7 (3)	C15—C14—C17—C20	5.0 (5)
N1—Pb1—S1—P1	−100.50 (7)	C13—C14—C17—C20	−176.8 (4)
N2—Pb1—S1—P1	84.2 (2)	C15—C14—C17—C19	125.9 (4)
S3—Pb1—S1—P1	170.20 (4)	C13—C14—C17—C19	−55.9 (5)
S4—Pb1—S1—P1	174.61 (5)	C15—C14—C17—C18	−114.4 (4)
S2—Pb1—S1—P1	−1.54 (4)	C13—C14—C17—C18	63.8 (5)
O2—P1—S2—Pb1	119.60 (10)	C25—N1—C21—C22	1.1 (6)
O1—P1—S2—Pb1	−131.28 (9)	Pb1—N1—C21—C22	−177.7 (3)
S1—P1—S2—Pb1	−2.37 (6)	N1—C21—C22—C23	−0.3 (6)
N2A—Pb1—S2—P1	−88.7 (4)	C21—C22—C23—C24	−0.5 (6)
N1—Pb1—S2—P1	84.95 (8)	C22—C23—C24—C25	0.5 (7)
N2—Pb1—S2—P1	−85.1 (3)	C21—N1—C25—C24	−1.2 (6)
S3—Pb1—S2—P1	−14.11 (8)	Pb1—N1—C25—C24	177.7 (3)
S1—Pb1—S2—P1	1.57 (4)	C23—C24—C25—N1	0.4 (7)
S4—Pb1—S2—P1	−175.44 (4)	N2A—Pb1—N2—C30	−177 (9)
O3—P2—S3—Pb1	−110.71 (10)	N1—Pb1—N2—C30	−44.2 (16)
O4—P2—S3—Pb1	141.59 (9)	S3—Pb1—N2—C30	−97.5 (10)
S4—P2—S3—Pb1	12.87 (6)	S1—Pb1—N2—C30	−15.7 (10)
N2A—Pb1—S3—P2	−100.2 (4)	S4—Pb1—N2—C30	−166.4 (10)
N1—Pb1—S3—P2	83.19 (8)	S2—Pb1—N2—C30	52.6 (10)
N2—Pb1—S3—P2	−104.8 (3)	N2A—Pb1—N2—C26	−25 (7)
S1—Pb1—S3—P2	169.87 (5)	N1—Pb1—N2—C26	108.0 (12)
S4—Pb1—S3—P2	−7.83 (4)	S3—Pb1—N2—C26	54.7 (11)
S2—Pb1—S3—P2	−175.46 (5)	S1—Pb1—N2—C26	136.5 (11)
O3—P2—S4—Pb1	113.52 (11)	S4—Pb1—N2—C26	−14.2 (12)
O4—P2—S4—Pb1	−138.86 (10)	S2—Pb1—N2—C26	−155.2 (12)
S3—P2—S4—Pb1	−11.90 (6)	C30—N2—C26—C27	−18 (2)
N2A—Pb1—S4—P2	92.0 (4)	Pb1—N2—C26—C27	−174.1 (9)
N1—Pb1—S4—P2	−80.48 (7)	N2—C26—C27—C28	8.8 (18)
N2—Pb1—S4—P2	91.1 (3)	C26—C27—C28—C29	0.9 (14)
S3—Pb1—S4—P2	8.03 (4)	C27—C28—C29—C30	0.2 (15)
S1—Pb1—S4—P2	3.37 (8)	C26—N2—C30—C29	18.7 (19)
S2—Pb1—S4—P2	177.68 (4)	Pb1—N2—C30—C29	171.6 (9)
O2—P1—O1—C1	175.0 (2)	C28—C29—C30—N2	−11.0 (17)
S2—P1—O1—C1	57.4 (3)	N1—Pb1—N2A—C26A	46 (4)
S1—P1—O1—C1	−74.6 (3)	N2—Pb1—N2A—C26A	119 (9)
O1—P1—O2—C11	−77.6 (3)	S3—Pb1—N2A—C26A	19.6 (17)
S2—P1—O2—C11	41.4 (3)	S1—Pb1—N2A—C26A	101.1 (17)
S1—P1—O2—C11	169.0 (2)	S4—Pb1—N2A—C26A	−49.7 (17)
O4—P2—O3—C31	74.6 (3)	S2—Pb1—N2A—C26A	169.0 (17)
S4—P2—O3—C31	−168.2 (2)	N1—Pb1—N2A—C30A	−128.3 (17)
S3—P2—O3—C31	−39.8 (3)	N2—Pb1—N2A—C30A	−55 (7)
O3—P2—O4—C41	176.0 (2)	S3—Pb1—N2A—C30A	−154.8 (13)
S4—P2—O4—C41	62.8 (3)	S1—Pb1—N2A—C30A	−73.3 (13)
S3—P2—O4—C41	−67.9 (3)	S4—Pb1—N2A—C30A	135.9 (13)
N2A—Pb1—N1—C25	−121 (2)	S2—Pb1—N2A—C30A	−5.4 (12)
N2—Pb1—N1—C25	−147.7 (9)	C30A—N2A—C26A—C27A	12 (3)
S3—Pb1—N1—C25	−94.6 (3)	Pb1—N2A—C26A—C27A	−162.0 (12)
S1—Pb1—N1—C25	−176.1 (3)	N2A—C26A—C27A—C28A	−7 (3)
S4—Pb1—N1—C25	−25.1 (3)	C26A—C27A—C28A—C29A	−1 (2)
S2—Pb1—N1—C25	116.7 (3)	C27A—C28A—C29A—C30A	2.1 (19)
N2A—Pb1—N1—C21	58 (2)	C28A—C29A—C30A—N2A	3 (2)
N2—Pb1—N1—C21	31.1 (10)	C26A—N2A—C30A—C29A	−11 (2)
S3—Pb1—N1—C21	84.2 (3)	Pb1—N2A—C30A—C29A	164.6 (9)
S1—Pb1—N1—C21	2.7 (3)	P2—O3—C31—C32	104.4 (4)
S4—Pb1—N1—C21	153.7 (3)	P2—O3—C31—C36	−78.1 (4)
S2—Pb1—N1—C21	−64.5 (3)	C36—C31—C32—C33	−0.2 (6)
P1—O1—C1—C6	112.6 (3)	O3—C31—C32—C33	177.3 (3)
P1—O1—C1—C2	−73.9 (4)	C31—C32—C33—C34	−0.2 (6)
C6—C1—C2—C3	−0.9 (6)	C32—C33—C34—C35	0.4 (6)
O1—C1—C2—C3	−174.3 (4)	C32—C33—C34—C37	178.9 (4)
C1—C2—C3—C4	1.3 (7)	C33—C34—C35—C36	−0.3 (7)
C2—C3—C4—C5	−0.4 (7)	C37—C34—C35—C36	−178.8 (4)
C2—C3—C4—C7	178.7 (4)	C32—C31—C36—C35	0.3 (6)
C3—C4—C5—C6	−0.9 (6)	O3—C31—C36—C35	−177.1 (4)
C7—C4—C5—C6	−180.0 (4)	C34—C35—C36—C31	0.0 (7)
C2—C1—C6—C5	−0.4 (6)	C35—C34—C37—C38	−176.2 (4)
O1—C1—C6—C5	173.1 (3)	C33—C34—C37—C38	5.5 (5)
C4—C5—C6—C1	1.3 (6)	C35—C34—C37—C40	63.1 (5)
C5—C4—C7—C10	−114.9 (5)	C33—C34—C37—C40	−115.3 (4)
C3—C4—C7—C10	66.1 (5)	C35—C34—C37—C39	−56.1 (5)
C5—C4—C7—C10A	−173 (3)	C33—C34—C37—C39	125.5 (4)
C3—C4—C7—C10A	8 (3)	P2—O4—C41—C46	97.9 (3)
C5—C4—C7—C8	126.8 (5)	P2—O4—C41—C42	−86.4 (4)
C3—C4—C7—C8	−52.3 (6)	C46—C41—C42—C43	0.7 (6)
C5—C4—C7—C9A	−36 (3)	O4—C41—C42—C43	−175.0 (3)
C3—C4—C7—C9A	145 (3)	C41—C42—C43—C44	0.2 (6)
C5—C4—C7—C9	5.0 (6)	C42—C43—C44—C45	−0.9 (6)
C3—C4—C7—C9	−174.0 (5)	C42—C43—C44—C47	177.2 (4)
C5—C4—C7—C8A	71 (3)	C43—C44—C45—C46	0.7 (5)
C3—C4—C7—C8A	−108 (3)	C47—C44—C45—C46	−177.4 (3)
P1—O2—C11—C16	−140.5 (3)	C42—C41—C46—C45	−0.8 (5)
P1—O2—C11—C12	41.8 (4)	O4—C41—C46—C45	174.9 (3)
C16—C11—C12—C13	−1.5 (5)	C44—C45—C46—C41	0.1 (5)
O2—C11—C12—C13	176.1 (3)	C45—C44—C47—C50	66.9 (5)
C11—C12—C13—C14	−0.2 (6)	C43—C44—C47—C50	−111.1 (5)
C12—C13—C14—C15	1.6 (6)	C45—C44—C47—C49	−171.8 (4)
C12—C13—C14—C17	−176.7 (4)	C43—C44—C47—C49	10.2 (6)
C13—C14—C15—C16	−1.3 (5)	C45—C44—C47—C48	−53.3 (5)
C17—C14—C15—C16	177.0 (3)	C43—C44—C47—C48	128.8 (4)

### Hydrogen-bond geometry (Å, º)

**Table:**

*D*—H···*A*	*D*—H	H···*A*	*D*···*A*	*D*—H···*A*
C12—H12···O1	0.95	2.45	3.083 (5)	124
